# Tracking Systems in Team Sports: A Narrative Review of Applications of the Data and Sport Specific Analysis

**DOI:** 10.1186/s40798-022-00408-z

**Published:** 2022-01-25

**Authors:** Lorena Torres-Ronda, Emma Beanland, Sarah Whitehead, Alice Sweeting, Jo Clubb

**Affiliations:** 1 Institute for Health and Sport, Victoria University, Melbourne, Australia; 2 Spanish Basketball Federation, Madrid, Spain; 3 Sports Performance, Buffalo Bills, Buffalo, USA; 4 Carnegie School of Sport, Leeds Beckett University, Leeds, UK; 5 Leeds Rhinos Netball, Leeds, UK; 6 School of Sport, Exercise and Rehabilitation, University of Technology Sydney, Sydney, Australia

**Keywords:** Global position, Optical tracking, Radio frequency systems, Technology, Performance

## Abstract

Seeking to obtain a competitive advantage and manage the risk of injury, team sport organisations are investing in tracking systems that can quantify training and competition characteristics. It is expected that such information can support objective decision-making for the prescription and manipulation of training load. This narrative review aims to summarise, and critically evaluate, different tracking systems and their use within team sports. The selection of systems should be dependent upon the context of the sport and needs careful consideration by practitioners. The selection of metrics requires a critical process to be able to describe, plan, monitor and evaluate training and competition characteristics of each sport. An emerging consideration for tracking systems data is the selection of suitable time analysis, such as temporal durations, peak demands or time series segmentation, whose best use depends on the temporal characteristics of the sport. Finally, examples of characteristics and the application of tracking data across seven popular team sports are presented. Practitioners working in specific team sports are advised to follow a critical thinking process, with a healthy dose of scepticism and awareness of appropriate theoretical frameworks, where possible, when creating new or selecting an existing metric to profile team sport athletes.

## Key Points

- Data from tracking systems can be used across a myriad of applications, which can be broadly grouped into describing, planning, and monitoring external loads, all with a view to supporting objective decision-making pertaining to performance and injury risk.
- It is advisable to be critical by considering precision (validity and reliability) and ecological validity when selecting from the multitude of metrics available in such systems, and when analysing time series derived from the data; selecting the most suitable information to a specific team sport, environment and playing position is also critical.
- Considering tracking data through the lens of a specific team sport reveals how the context and constraints (e.g., playing dimensions, player density, position characteristics, game rules, timing structure, physical demands, among others) of a sport influence how such information can be applied. The alignment of technical-tactical and physical data provides practitioners with greater context for the physical characteristics, and perhaps greater application of tracking data to training practices.

## Introduction

Athlete tracking systems have become commonplace in professional team sports. Seeking to obtain a competitive advantage, organisations are investing financial and time resources in technologies that can quantify training and competition characteristics in a valid and reliable manner. It is expected that such information can support decision-making processes for the prescription and manipulation of training load [1].

External load has been described as the foundation of a monitoring system [2], and is represented by the activities performed by an athlete [1]. As part of the monitoring process, tracking data are used to quantify external load. Tracking data can be combined with other streams of information to determine readiness for competition, analyse the load-performance relationship, support appropriate planning for training and competition load, as well as to minimise risk of injury, illness and non-functional overreaching [2]. To enable such applications, it is advised to integrate external load in a multivariate monitoring system that may include internal load—the physiological stress imposed by the external load—and training response mechanisms [3]. Therefore, the appropriate collection and interpretation of tracking data is vital for this process.

The description, planning, and monitoring of external load provides valuable information for understanding the training and competition characteristics of various team sports. As interest in this information has garnered greater attention, manufacturers also attempt to improve their filtering systems and algorithms by incorporating new variables to satisfy the needs of their consumers. However, this rapid improvement and rollout may lead to confusion between different sports whereby, with differing needs, the user has to carry out a critical thinking process to guarantee the optimal use of tracking systems and subsequent data, within their own context. As outlined by Torres and Schelling [4], the implementation of technology in the applied setting should be driven by recognising a suitable solution to a problem in the specific environment. With a plethora of external load metrics and methodologies constantly emerging [5–7], it is paramount to understand how the data can be analysed in order to add objectivity to decision making and to ultimately support the athletic training process.

Once the characteristics and limitations of different tracking systems used in team sports [Global Positioning Systems (GPS); Optical Tracking; Local Positioning Systems (LPS); and Inertial Measurement Units (IMU)] are understood, there is a need for a pragmatic and systematic approach to data collection, analysis and interpretation.

In this narrative review, we present practical applications for data provided by tracking systems. Reviewing all aspects of a monitoring system is beyond the scope of this narrative review, which focuses on tracking systems only. Whilst research has typically focussed on specific methods of analysing such data, the literature lacks an overview of the various purposes of tracking data in relation to the context of specific team sports. Therefore, the objective of this review is to examine the critical thinking required to select the most suitable metrics and describe the varying evidence-based applications of tracking data in the applied setting. We then aim to demonstrate this critical process by discussing the specific considerations and contexts of analysing tracking data within the context of seven different team sports.

## Tracking Metrics

The metrics provided by tracking technologies may vary between systems. For example, optical tracking determines 2-dimensional coordinates that can be extrapolated into distance and speed measures, whereas IMU combine data from multiple sources (e.g., accelerometer, magnetometer, and gyroscope) to measure acceleration of the body or body segment. Some technologies also combine multiple tracking systems, such as GPS, LPS and IMU, within a single device [8]. Certain professional sports, and relevant governing bodies, do not permit the use of certain systems within competition (e.g., National Basketball Association; NBA). Hence, practitioners may be required to combine metrics from different systems to integrate data across training and competition. As such, understanding which metrics are provided by the different systems, their definitions, calculations, ecological validity and specificity use to a sport (or team, athlete), is paramount to the practitioner.

### Considerations for Metric Selection

Practitioners are besieged with a multitude of metrics from tracking systems (Table 1). Categorising these metrics according to their similarities may be adequate in appraising their usefulness. Distances covered at various speeds and the occurrences of high-speed movement, accelerations and decelerations (Levels 1 and 2; Table 1) appear to be those most commonly reported by practitioners in team sports [9, 10]. However, this does not mean they are necessarily appropriate in all sports. Selecting the most pertinent metrics, given each sport’s unique constraints, is vital to ensure metrics are appropriate for the context of a specific sport.

**Table 1:** Definitions of common tracking metrics

Level	Metric	Definition	Common Measures
1	Distance	Cumulative distance	Total, Relative, Distances in speed/acceleration/deceleration zones
2	Acceleration (2D)	Instantaneous peak rate of positive change in velocity	Maximal/Peak, Average, Distance/Efforts/Time in acceleration zones
	Deceleration (2D)	Instantaneous peak rate of negative change in velocity	Maximal/Peak, Average, Distance/Efforts/Time in deceleration zones
	Change of Direction (2D)	Count and intensity of changes of direction derived from positional data	Total, Percentage Difference Left *vs* Right, Count in intensity zones
3	Accelerometry-derived load	A manufacturer-specific, modified vector magnitude of 3D acceleration values (expressed in AU)	Total, Relative to time, Relative to distance, 2D (excludes vertical axis), 1D (absolute or relative contribution of individual axes)
	Change of Direction (3D)	Count and magnitude (g) of changes of direction derived from inertial sensors	Total, Percentage Left v Right, Count in intensity zones
	Impacts	A manufacturer-specific metric that provides a count of 3D acceleration values (g) over a threshold	Count and Magnitude of Impacts
	Collisions/ Tackles	A manufacturer-specific metric that classifies collisions specific to the sport	Count and Magnitude of Collisions
	Stride Variables	Accelerometry-derived metrics estimating ground contact time	Contact Time, Flying Time, Vertical Stiffness (KN·m)
	Stride Imbalances	Accelerometry-derived metrics split by left and right side	Percentage Left v Right
Hybrid	Speed	Instantaneous peak rate of position change	Maximal/Peak, Average
	Sport-specific Metrics	Specific machine learning algorithms designed to quantifying movement demands per sport	AMF QB throws, Basketball court transition, IH skating strides, Rugby scrum detection, Soccer GK Left v Right Dive Count
	Metabolic Power	Estimates the energetic demands of high-intensity Level 1 and 2 actions via GPS or LPS data	Metabolic Energy (Cal·kg), Equivalent Distance (distance covered running at constant speed on flat terrain, for a given energy expenditure), Total Metabolic Power (ml·kg·min), Distance/Efforts/Time in Metabolic Power bands

Level 1: distances covered in different velocity zones; Level 2: events related to changes in velocity (i.e. acceleration, deceleration, and changes in direction); Level 3: events derived from the inertial sensors; Hybrid = combination of levels (28)

2D, 2-dimensional; 3D, 3-dimensional; AU, arbitrary units; g, g force; kN, kilonewton; m, metre; AMF, American Football; IH, Ice Hockey; GK, Goalkeeper; Cal, calorie; kg, kilogram; ml, milliliter; min, minute

In the applied sport environment, there is a need to distil the myriad of metrics into concise, meaningful information. Decision makers (e.g., coaches and performance staff) require simple, accurate, and coherent feedback, including succinct conclusions from data in a timely manner [11]. It is advisable to include stakeholders in the metric selection process, as coaches have expressed an interest in tracking data pertaining to ‘high-intensity’ actions and ‘intensity’ [12]. Indeed, greater involvement and improved communication in this process could help to avoid a detrimental gap between information and its impact [13].

**Fig. 1:**
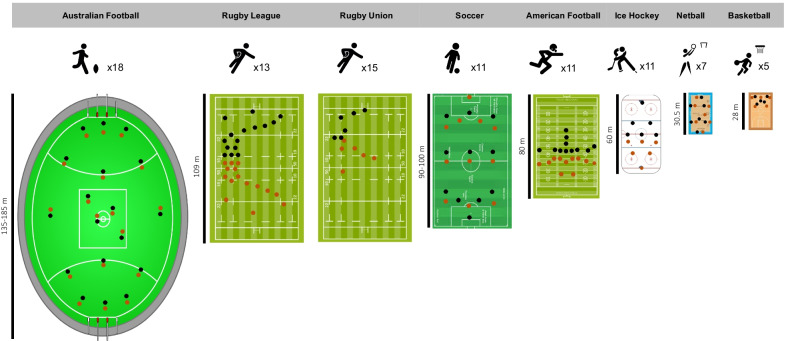
A comparison of field size across different team sports. The number of players per team is represented by the figure above each field. The numbers of players, team and opponents number, are also represented by the dots shown on the field (not to scale). Orange and black colours represent opposing teams

**Fig. 2:**
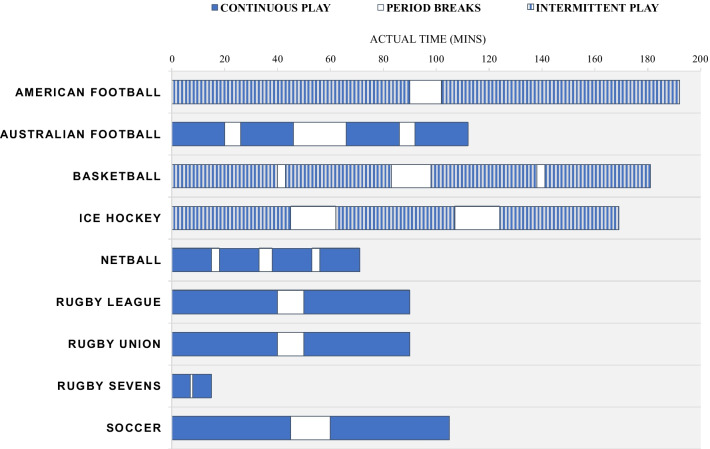
A bar chart visualising the difference in playing time and actual time during match-play across different team sports. Continuous play is shown in solid blue. These sports may have pauses in play for substitutions but do not have pauses in game-play periods for commercial breaks or time-outs. Sports of a play-by-play nature (clock stop), with intermittent breaks, for example for faults or commercial reason, are shown in striped blue and grey

A variety of considerations for selecting metrics are shown in Figs. 1 and 2. These include, but are not limited to: playing dimensions, player density, position characteristics, game rules, and timing structure. The combination of these factors demonstrates the importance of context both between and within sports. As an illustration, it could be questioned if tracking an athlete’s maximum velocity is pertinent in basketball, given the limitation of court size. Similarly, absolute high-speed running (HSR) in American football may be much less meaningful to linemen than wide receivers due to their unique positional characteristics (see Sect. 4). However, this process presents somewhat of a paradox; the practitioner may first have to measure the characteristics to determine its meaningfulness and then be able to apply critical thinking in order to determine the most appropriate measures to the sport.

Subjectivity is important in the critical selection of metrics, based on the sport and/or position characteristics. However, despite a more subjective process based on observation, it remains that such metrics should still demonstrate face validity (perceived to be relevant) and high content validity (representative of the construct, in this case external load) —for further information, interested readers are directed to [14]. There may be scope to further support this process with objectivity via statistical analysis of the existing suite of metrics available. Weaving and colleagues (2019) demonstrated how principal component analysis, a linear algebra technique, can reduce and visualise complex tracking data into meaningful components [15]. This approach can help the practitioner to understand multi-collinearity between metrics and therefore, filter out redundancies [15]. For example, in professional rugby league, such analysis suggested some measures of training load (e.g., training impulse, session rating of perceived exertion, body load, high-speed distance, and total impacts) may be used interchangeably to describe small-sided game and conditioning loads, but not for other modes such as skills, wrestling, and speed training [16]. Similarly, by using feature selection in the time and frequency domain, team sport related activities can be accurately classified through a single tracking device. This approach may allow for the generation of more sport and activity specific algorithms, from one device [17]. Such approaches may be especially worthwhile, given that a multivariate approach to monitoring has been recommended for team sports [16]. Focus should therefore be given to developing and improving metrics already in use, ideally through the integration of physical and tactical insights into combined metrics [18]. The onus is thus on the practitioner, to determine the most relevant metrics to interpret and communicate with key stakeholders.

The workflow of selecting the most pertinent tracking metrics is an ongoing process, requiring current knowledge on the validity and application of tracking technologies, based on research and technological advances. Recently, the measurement and management of decelerations have been declared important to capture, given their distinct demands and potential as a critical mediator of neuromuscular fatigue and tissue damage [19]. However, concerns have been raised regarding the precision of tracking devices to capture accelerations and decelerations [20]. Whilst sport and tracking technologies are constantly evolving, practitioners need to balance innovation with an understanding of the precision and sensitivity of technology.

## Applications of Tracking Data

**Fig. 3:**
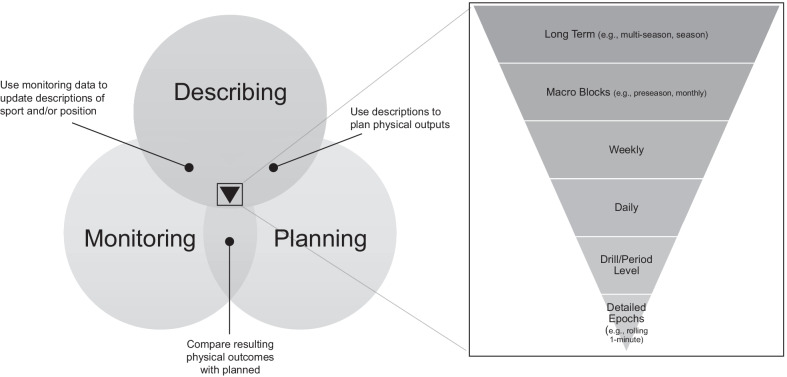
Applications of tracking systems data. The overlapping purposes of Describing, Planning, and Monitoring are shown. The inverted Reuleaux triangle in the centre of the Venn diagram represents the varied time analysis approaches, drilling down by time, that can be applied

Once the precision and accuracy of a tracking system are quantified, attention can turn to the analysis process. Tracking data can be used to identify key competition characteristics, including the most demanding situations, in order to objectively manage physical preparation, readiness, and return-to-play. Buchheit and Simpson (2016) proposed three main objectives for tracking data to: i) better understand locomotor characteristics and external load; ii) assist the programming of team training external load; and iii) help with decisions pertaining to performance and injury risk as they relate to an individual’s programme [11]. These objectives can be condensed into the following overarching and overlapping purposes: Describing, Planning, and Monitoring (Fig. 3).

### Describing

Descriptive studies are an essential first step in epidemiological research [21]. This is reflected in applied sport science, whereby the initial application of a tracking system is to quantify locomotor characteristics across different contexts. The first notational systems in the 1970s were used to describe the differences in external load across playing position in football match-play [22]. Since then, training and competition outputs have been described by playing position in a variety of team sports [23–26]. Given the growing availability of tracking systems in youth environments, such descriptions now also extend across age groups [27] and bio-banding, according to maturity status [28].

Given that tracking systems have existed for more than four decades, calls have been made to further descriptive analysis. One proposal is to adopt an integrated approach to competition tracking data that contextualises characteristics by combining physical and tactical data [18]. This alignment of different data sources may allow for an improved understanding and translation of training to performance in team sport [18]. Spatiotemporal data, integrated with tactical context, have allowed the exploration of new concepts, including space occupation [29], off-the-ball scoring opportunities [30], the risk-reward of passing [31], and team pace of play [32]. These analytical approaches have started to delve into the complex problems that sport scientists face, and go beyond simply describing aggregate external load data. With the rise in data availability, affordability and accessibility, sport scientists now have the opportunity to apply many analytical techniques to the same tracking dataset, thus expanding upon descriptive reporting.

### Planning and the Intersection with Describing

Practitioners use descriptive data to aid in the planning of training. Theoretical frameworks of the training process depict how external load, as determined by the training plan, is prescribed to elicit the desired training outcomes [33, 34]. Training plan development involves combining both an objective (e.g., external load) and subjective (e.g., coach experience) understanding of the sport’s characteristics. In team sports, performance is complex and the training process involves more consideration than physical inputs alone. However, it remains that fundamental training principles, such as overload and progression, should form the basis of physical preparation and training design [34].

A sliding scale of timeframes can be considered when planning the training process; long-term, seasonal, and day-to-day planning may all incorporate objective information provided by tracking systems [35]. This may be especially pertinent during periods of congested fixtures. For example, netball at the elite level is played across tournament style competition during World Cups and Commonwealth Games. This congested schedule, with matches often played twice per day, can result in reduced wellbeing markers, sleep quality and neuromuscular function [36]. Similarly, in field hockey, daily wellbeing markers were accompanied by a reduction in HSR, despite rest days [37]. Collectively, these results demonstrate the importance of a multivariate monitoring system whereby, both dose and response are tracked.

Higher external loads have been demonstrated during preseason training and yet, preseason participation may help to protect players from injury in the regular season [38]. According to training theory this can be expected, given it is the systematic repetition of a stimulus and its associated response that are necessary to elicit chronic adaptations [33]. Training plans, attempting to optimise this systematic repetition of stimulus, can be generated from tracking data using a constrained optimisation framework, to optimise physical performance and reduce injury-risk. Specifically, practitioners can use machine-generated algorithms to hone-in on how much risk is associated with a particular external load, by adjusting metrics or levers including total distance or HSR, for example. As an example, in Australian Football [39], Banister’s impulse-response was utilised for the training load—risk model [39], however, the same conclusions can be delivered from other studies in other sports using the most pertinent parameters to their environment [38]. Whatever method utilised, clear communication between practitioners and coaching staff is advised, to align and iteratively review physical and tactical objectives throughout the planning process, given that communication quality between coaching and medical staffs has been shown to be associated with injury burden and player availability [40].

Training goals within cycles are varied in an attempt to balance physical preparation and readiness for competition. For instance, it is commonplace for team sport training one day prior to competition to be substantially lower in external load than others within the microcycle [41, 42]. As the days between competitions increase, training load will also increase [43]. Therefore, the opportunity to apply tracking data analysis to influence planning may vary across and within sports, depending on fixture congestion. For example, American football schedules one game per week whilst soccer can have one or two, and basketball or ice hockey face three or four games per week during the in-season phase (see Sect. 4).

Competition characteristics are used as a benchmark to understand the most intense periods of play, from which the design of appropriate drills replicating or surpassing the intensity of the game can be planned [44]. Indeed, drill design can have a substantial impact on the external load elicited and thus is a vital piece of the planning process [45]. This is illustrated through research into small-sided games in soccer, whereby the number of players, floating players, pitch size, rules, goalkeepers, duration of bouts, and coach encouragement each impact external load [45–47]. By quantifying and storing drills in a systematic way, a database can be utilised to analyse and subsequently plan drill rules, duration, sets and repetitions structure, amongst other considerations. Armed with this objective information, practitioners could support coaches with a training design that elicits physical outputs in line with the training goals, whilst also respecting other training objectives outside of the physical realm (e.g., tactical, technical, psycho-social, cultural).

Utilising descriptions of training and game characteristics also assists with planning for the rehabilitation process. Returning a player to competition after injury is a challenging and complex process, which involves balancing risk and objective criteria with subjective experience [48]. Tracking data can therefore assist in the planning of rehabilitation from control to chaos, designed to meet the individual needs of the sport, playing position, individual athlete, and the specific injury in question [49].

### Monitoring and the Intersection with Planning

Planning is an essential part of the training process, whereby tracking system data can play a vital role. However, for plans to be successful, it is advisable for stakeholders to be aligned and communicate physical training goals, before, during and post, the monitoring of training and rehabilitation. This is typically conducted as part of an ongoing review process across the multidisciplinary performance staff. The monitoring process has two main purposes: i) to assess the interaction between the resulting external loads compared with those that were planned [34], and ii) to analyse the dose–response of said training loads on a team and individual basis [35]. The data derived from tracking systems can be key for this process, providing practitioners with vital information on an athlete’s external load.

Performance staff plan the external load for desired training adaptations and responses from a drill to macro-cycle level (Fig. 3) to assist in performance, development and injury risk reduction [11]. As discussed, practitioners put plans in place to target an appropriate volume and intensity of training, at the right time in the training cycle, to either increase or decrease fatigue [2, 50]. In team sports, one of the goals of tracking systems data is to assess whether athletes have been subjected to the planned training load [34]. This can be accomplished through live monitoring and retrospective session analysis. The first, live monitoring, enables in-session adjustments to assist with trying to achieve the planned load during the training session [35], and ultimately contribute to the chronic fatigue-recovery response.

Training load management approaches have been widely researched and utilised in an attempt to reduce the risk of injury [51]. However, the ability to control the risk of injury, through the manipulation of training load, has recently come under scrutiny [34]. This is due to methodological concerns in the analysis of training load data [34]. Whilst this specific topic is beyond the scope of the present review, it remains that understanding the external load quantified through tracking system data is a useful tool as part of training load planning and monitoring processes [35]. An understanding of the external load placed on the athlete(s) can assist in titrating the fatigue response [2]. Further, it has been recommended that practitioners should not focus on external load alone [33]. The quantification of internal load, and the response to training, should be considered as part of a multivariate system alongside the external load data, in order to understand the dose–response relationship to training [16]. Including measures of the athletes’ individual characteristics, such as fitness [52]or maturation [28], into such a system also merits consideration. The cycle of planning and monitoring is an ongoing, iterative process in which the quantification and evaluation of planned and implemented load alongside training responses and outcomes can be beneficial to practitioners and the coaches and athletes they strive to support.

### The Intersection of Monitoring-Describing and the Sport Evolution

The physical characteristics of sport evolve over time and thus, another application of tracking is to use ongoing monitoring data to update the objective description of the sport. For example, HSR has evolved in the Premier League (soccer), with a 35% increase in sprint distance over a seven-season period [53], a trend that has an even greater impact on full backs (36–63% increase) [54]. Evolving physical characteristics have also been demonstrated across other team sports [55, 56]. Similarly, longitudinal changes in physiological profiles may be representative of changes in competition characteristics, which could be explored with the use of tracking technology [57]. Such findings have implications for physical preparation and highlight the need to update objective descriptions of physical characteristics with ongoing data collection.

The evolution in physical characteristics within a sport may, in part, be influenced by changes to the rules of the game, which may be captured by ongoing monitoring of tracking data. For instance, rule changes that reduce the time taken to restart play in men’s professional Australian football have led to an increased flow and speed [55]. Changes to the kickoff portion of an NFL game, including a stationary start for coverage players, may have resulted in a change to physical outputs that contributed to a notable decrease in concussion injuries according to league medical officials [58]. The combination of IMU, pressure sensors and video cameras have been used to assess biomechanical loading in multiple scrum engagement techniques in rugby, with a pre-binding technique shown to reduce the stresses acting on the players [59]. The use of mouthguards instrumented with accelerometers and gyroscopes is also being explored in collision sports for measuring head kinematics, with a view to assisting with the detection and monitoring of concussion [60]. Such examples highlight the need to update the quantitative description of a sport as new means of monitoring become available. Clearly, sport is a dynamic ecosystem that changes over time. Therefore, tracking data allows the ability to recursively describe, plan, and monitor external load in line with a specific sport’s characteristics.

### Between-System Interchangeability

In order to provide complete athlete monitoring, practitioners often need to combine tracking data from multiple systems [61]. Understanding the agreement between systems is important for practitioners, in order to track meaningful changes in profiles [62]. Comparison of optical tracking to GPS has shown slight-to-moderate and moderate-to-large differences for total distance and HSR distance (> 18 km∙h), respectively [63]. Buchheit (2014) found trivial-to-small overestimation of distance (5.4%) and slight-to-moderate overestimation of HSR (> 19.8 km∙h: 26.5%). These differences highlight the importance of considering GPS sampling rate, the number of visible satellites connected, satellite signal strength, and software filtering when reviewing system comparisons [64]. Recent advances in GPS hardware technology have resulted in a stronger correlation with an optical tracking system [61, 65]. Given that such differences remain, a recent area of interest is the use of predictive equations to account for system differences and enhance accuracy of the interchangeability of data [60, 61]. A number of techniques can be used to assess interchangeability between systems, including regression analysis.

### Time Series Analysis

There are many applications and approaches to time series analysis of tracking data throughout the processes of Describing, Planning, and Monitoring, as indicated in Fig. 3. To determine an appropriate approach, practitioners and researchers can consider the most relevant time analysis approach(es) according to their specific sport, setting and intended application of the data. Reporting metrics derived from tracking systems can be done in a variety of ways, including by absolute values, temporal durations, moving averages or as normalised data (e.g., per 100 h played, per 100 possessions). Absolute values often describe metrics per the whole match, halves/quarters and training periods. Such aggregated approaches are commonly used across the literature in a range of sports, providing an indication of the external load encountered by athletes [66, 67]. This information can be useful in practice, providing total volumes and averages to assist in training planning and periodisation, especially when combined with internal load measures [68].

However, aggregated values are limited in the prescription of specific training practices given the intermittent nature of team sports. Therefore, practitioners may also consider other time -analysis approaches. Match-play and training can be stratified into periods based on different temporal durations (e.g., 5-min or 10-min) to capture the fluctuation in external load throughout match-play [69–71] and the peak characteristics, sometimes known as the “worst-case scenario” [72]. A segmental approach (e.g., the match file is split from zero according to the duration: 0–5 min, 5–10 min), a moving averages approach (i.e., a rolling average of the raw instantaneous data) [72], or time series segmentation (i.e., the computation of non-uniform segments from a time series) can also be used [73]. Additionally, the physical characteristics of match-play can be stratified per phases-of-play or by match-activities through the alignment of video and tracking systems data. Examples of such approaches in the current literature include: stratification per attack and defense [74, 75] or per possession of the ball [76, 77]. The alignment of technical-tactical and physical data provides practitioners with greater context to the physical characteristics, and perhaps greater application to training practices [18, 78].

#### Period Selection

The selection of the period for analysis (e.g., whole-match, temporal duration or phase-of-play) should be determined by the primary use of the data. When stratifying match-play based on temporal durations, a range of durations have been utilised [72]. It is important to consider that when using temporal durations, the intensities will differ depending on the epoch analysed [25, 79] and data cannot purely be extrapolated for different epochs. If the intended use of the data is to aid in the prescription of training drills, this should drive the duration window analysed, however this is often not known when the analysis of match-play is carried out. The use of the power-law relationship with moving averages was therefore proposed, providing an equation to predict the peak intensities as a function of time [80], which has been utilised in research in a number of team sports [26, 80–83]. Similar approaches have recently been applied to model the decrement in peak acceleration magnitudes in basketball [84]. Such approaches can be used as a simple monitoring tool, in practice, without the pre-determined selection of an epoch. For example, the unique power-law relationship can be determined for a sport, team, squad and/ or competition, and then the peak intensity for the desired time frame can be predicted from the equation. This approach can also allow practitioners to further investigate the peak periods of activity during training and matches, from a continuous data trace.

#### Peak Demands

The quantification of the peak passages of match-play has gained popularity in recent years, due to the practical utility of the data over whole match/ training aggregated values [25, 44, 85] and the availability of raw trace data. Research has quantified the peak locomotor demands (sometimes described as the “worst case scenario”) of match-play across the football codes [72], and other field-based team sports (e.g., lacrosse [85], field hockey [26] and court-based team sports [e.g., netball [81], basketball [84, 86]). Whilst different methodologies have been utilised—including segmental or moving averages and ball-in-play [44]—the moving averages approach is the most commonly used [87–90], given its ability to capture the subtle fluctuations in the intensity of match play, as well as the functionality of the power-law relationship. An example of its use is the monitoring of the intensity of small-sided games, to attempt to replicate for the intensity of peak periods of match-play [45]. Through the use of live monitoring, the intensity can be monitored and manipulated via feedback to coaches and consequent alterations to the match-play for example rules, pitch size and player numbers [35].

However, further considerations should be made regarding the depth in analysis of the peak demands. To shift the focus from one metric in isolation and enhance training application, the quantification of the concurrent demands within the most demanding physical passages of play can be investigated. For example, in collision sports (e.g., the rugby codes), the number of collisions that occur within the peak running periods, or the running that occurs within the peak collision periods, can be identified [3, 45, 91]. Additionally, it is important to understand the technical and tactical requirements alongside the physical data provided by tracking systems [18, 78], as well as any influence of contextual factors [92], to provide greater context to the data. Changes in the peak movement demands in relation to skill involvement have been investigated in Australian football, highlighting reductions in the movement profile as the number of involvements increases [91]. Additionally, in soccer, the “worst-case scenario” has been found to be impacted by contextual factors (i.e., match-location, match-outcome) [93], with greater peak characteristics in away games compared to home games, or the effect of ball possession within these worst-case scenario periods [18]. Moreover, it has been recently shown that the “worst-case scenario” produces unstable metrics that lack context, with high variability, and therefore, training drills targeting this metric may not have representative designs and so may underprepare athletes for future match demands [94].

#### Time Series Segmentation

Examining the physical output of team sport athletes via aggregate parameters has many challenges. Periods where physical output changes over time are unlikely to be detected when examining only the total distance covered or percentage of time spent performing high-intensity running. As described above, team sport athletes often execute periods of physical output whereby intensity is far greater than that of an averaged total game [25]. Given the volatility of team sport matches to identify meaningful changes on a per-second basis, moving minute intervals have been used to detect these periods [95]. However, the length of these moving intervals is often decided arbitrarily and typically only focuses on the rolling average (or peak average) of a metric [80]. Alternatively, time series segmentation involves detection of the mean and variance of a metric, over segments of non-uniform size, without the need for a priori defined intervals [73].

Time series data, including raw GPS and LPS traces, are characterised by their continuous nature, as opposed to match events which are transactional and discrete. Sport scientists are often faced with a difficult problem in *how* to analyse this continuous data, in order to derive meaningful information. A method which may be useful, when dealing with continuous data, is time series segmentation. This is an analysis technique that comprises of algorithms which search for change points within temporal data [96]. These change points designate that the pattern of subsequent data points is characteristically different to those prior [97]. Segments are automatically detected, based on a given number of change points, within an underlying time series, for example a raw GPS trace. This trace could be analysed via time-series segmentation, to detect how athlete physical output changes during a match, as a function of time. In team sport, time series analysis has been utilised in Australian football to identify and describe the segments of physical (and skilled) output during matches [73]. Similarly, time series analysis has been utilised to profile the skilled output in team sport matches [98] and predict team success in the English Premier League [99]. The visualisation of metrics from athlete tracking systems, including raw trace data that can be analysed via time series segmentation, requires the visual encoding of thousands of data points. Sport scientists thus need to decide whether to aggregate specific time periods (e.g., distance covered during an on-field rotation or stint), or all data points that are contained within the time period. Therefore, communicating how data are selected and analysed before visualisation occurs is an important skillset for the modern sport scientist.

## Sport Specific Analysis

Context will drive what technology (and in turn, metrics) should be selected to capture the characteristics of team sport athletes during training and competition. For example, in basketball and netball, the use of GPS is rendered inoperable, given at the elite level both sports play and train indoors. Therefore, LPS, IMU and optical tracking are more appropriate. Similarly, the use of optical tracking to monitor athletes during Australian football and rugby codes may be limited, given the large (and varying) field sizes, whereby many cameras would need to be installed at height around the ground. Therefore, the tracking technologies and derived metrics used for specific sports and playing positions need careful consideration. Below we have arbitrarily selected team sports and introduced sport-specific considerations that practitioners should be mindful of, when selecting the technology and corresponding metrics to profile the physical characteristics of athletes during training and matches.

### American Football

American football is an intermittent, contact sport characterised by physical demands that include HSR, accelerations, decelerations, and changes of direction [100]. The game is play-by-play in nature across four 15-min quarters, with multiple stoppages and commercial breaks, extending the game length, in actual time, to upwards of three hours (Fig. 2). Players are selected from a roster of 53 to 120, depending on the time of the season and the level (i.e. collegiate vs professional) with specialist positions across defense, offense and special teams [101]. Factors that set this sport apart includes the vast differences in positional characteristics, the mandatory inclusion of personal equipment (i.e., helmets and pads) that in turn likely influences the magnitude of collisions, and the prolonged time course over which the game is played. As such, there are nuanced considerations for applying tracking data in this sport.

The wide disparity of positional characteristics in this sport provides practitioners with challenges related to both physical preparation and tracking itself. The process for selecting metrics may be especially pertinent given that the notable difference in positional characteristics may lead to the focus of different metrics for different positions. Differences in running, assessed via HSR, and non-running, assessed via total inertial movement analysis (IMA) from the IMU, characteristics were notable across position groups during a professional training camp [102]. Similar differences have been illustrated in training and competition characteristics at the collegiate level [100, 103, 104]. While the use of IMU data may help to capture sport-specific actions (e.g., throwing, contact, and collisions) and be developed into position-specific metrics [105], this technology may still be unable to fully quantify some characteristics that rely less on movement tracking, such as the high isometric demands of grappling and blocking. Further, IMU technology is not permitted in competition at the professional level, wherein Radio Frequency Identification technology is currently employed [106].

Given the heterogeneity of the physical characteristics by position, relative velocity thresholds may be pertinent. Ward and colleagues (2017) used a HSR threshold above 70% of the maximum speed for the respective position group, derived from training sessions within the previous year. Absolute speed zones for the entire team, which may over- and under-estimate demands for faster and slower athletes respectively [100], have also been utilised. However, it is also important to note that research in other sports (soccer) found the use of relative speed thresholds did not better quantify the dose–response and, in fact, the application of a player’s peak speed to establish speed zones may result in erroneous interpretations [107]. More research is required in American football to determine the most suitable approach for quantifying the dose–response relationship, especially given the wide heterogeneity of characteristics by positions and also the variation of intensities within position-specific periods in a training session [102].

The heterogeneity of American football characteristics is exacerbated by the *special teams* element. During these passages of play, a mixture of offensive and defensive players (generally non-starters) combine to perform roles in support of specialist kickers, who are attempting punts, kickoffs, and/ or field goals. Thus, practitioners are challenged to prepare these players for the physical characteristics of both their primary and special teams roles concurrently. For example, a Linebacker who is also a special teams specialist, may play across all four phases of Punt, Punt Return, Kickoff, and Kickoff Return. If an injury occurs, the planned roles may be further influenced. These passages of play may often be the most physically demanding with regards to HSR (unpublished observations), and so there are repercussions for tracking the physical outputs of these passages, both in terms of understanding the specific characteristics and monitoring the external load each individual player is subjected to.

There may be further disparity in the physical requirements for players within numerous periods of a training session. Whilst a session may be divided into five key periods (i.e., warm up, position-specific training drills, special teams drills, preparatory plays, and team periods [102]), players may be required to work on different characteristics during those time periods, based on their role. For instance, starters not involved with special teams may be training separately according to their position role on offense and defense during such periods. This is an important contextual note for practitioners attempting to categorise, analyse, store, and plan periods/drills using a database.

Considering the physical characteristics of a session, period or individual play level, is worthwhile in the planning process, as American football is a sport characterised by a high tactical demand. With an intermittent play-by-play structure (Fig. 2), players are expected to learn set movement demands outlined in a playbook, more akin to set pieces in other sports. As such, certain time epoch analysis, including segmental analysis, rolling averages or game speed approaches, may be less relevant to track in this setting. Rather, tracking outputs on a specific play level may be more pertinent. Given the prominence of the integrated combination of physical, tactical, and technical characteristics of the game, there may be benefit in aligning tracking data with video and play/scheme notations to understand the physical outputs within the game context. Indeed, machine learning techniques are exploring the ability to classify route combinations, blocking assignments or coverage type from tracking data [108].

### Australian Football

Australian football is an invasion-sport contested between two teams of 22 players, 18 permitted on the field and four on the interchange bench. A unique constraint of the sport is the non-uniformity of field size. The dimensions of fields used within the professional competition, the Australian Football League (AFL) vary from 175 m in length and 145 m in width (University of Tasmania Stadium) to 155 m by 136 m (Sydney Cricket Ground). The average length and width of AFL grounds are 163.6 ± 5.9 m and 132.1 ± 6.9 m, respectively. One AFL field (Marvel Stadium) is indoors. Collectively, field size and stadia constrain the type of tracking systems used. In Australian football, GPS is commonly utilised during matches and training [109–111]. Given their suitability across outdoor and indoor stadia, inertial sensors including accelerometers, are also used [112]. Only recently have LPS been utilised during elite competition [113]. Using optical tracking is unsuitable for this sport, given the vast ground sizes that require a large number of cameras be used [114]. Athlete tracking systems are therefore, largely confined to accelerometers, GPS and LPS, and their derived metrics. The selection of which metrics to use, for the purpose of profiling Australian football training and match play, from these different systems is an important consideration.

The physical characteristics of these athletes is complex, part of interacting sub-systems and often reactive to a stimulus, including the ball, umpires, opponents or teammates. Understanding how these stimuli impact physical output is useful, to decide which metrics are meaningful. Features including anthropometric (e.g., height) and physiological (e.g., aerobic capacity) may impact external load. For example, aerobic fitness has a large effect on relative total and HSR distances covered during AFL matches [109]. Rucks in Australian football are typically taller than their teammates but cover up to 45% less distance at high-speed [109]. Environmental factors also impact metrics obtained. These results demonstrate that sport scientists should be mindful of the performer constraints during training and matches, which can impact the metrics.

A number of contextual factors influence the external load of Australian football athletes during training and matches. The number of rotations, margin, opposition quality and stoppages all impact the direction and magnitude of physical output in men’s matches [109]. In women’s matches, physical output is influenced by on-field rotation stint, opposition quality and margin [115]. Other contextual factors, including stoppages or brief breaks in play, also impact Australian football athlete external load. In elite men’s matches, increased stoppages result in less relative total distance covered [116]. Sport scientists should therefore be mindful of these contextual factors when analysing men's and women’s tracking data.

The relationship between physical and skilled characteristics has been examined in Australian football, in an attempt to give further context to physical metrics. Trivial and weak relationships exist between aggregated physical (e.g., absolute total high-intensity running) and skilled (number of involvements, including handballs and tackles) characteristics, when analysed via generalised linear models and conditional inference trees [113, 116]. Linear mixed models had low explanatory power whilst the conditional inference trees also had poor accuracy [113]. This is likely due to subtle changes in athlete physical and skilled output not detected in aggregate parameters. Moving averages have been utilised in Australian football, with men’s match intensities peaking at 223 ± 35 m^.^min across one-minute moving averages [117]. However, time-series analysis as described in 3.6.3 above, removes the need for manually selecting pre-defined time windows and can utilise the mean and variance of a metric. Athlete velocity data can then be examined, without having to rely on fixed duration windows, allowing for the detection of precisely when a peak match intensity occurs at a specific point in time [73]. By utilising time series and data mining techniques, sport scientists can therefore delve beyond aggregate parameters and extract features from raw GPS or LPS data. The specificity of Australian football training drills to matches could be examined by visualising the distribution of features from raw velocity traces, identifying when players obtain match intensities and how often this happens.

Given the abundant data available from athlete tracking systems and the dynamic, non-linear nature of the sport, sport scientists should look to move beyond reporting aggregate parameters, including total distance covered per drill, on-field rotation, quarter or match. Instead, sport scientists could utilise the raw velocity (or accelerometer) trace data to identify where, when and *how* Australian football athletes’ external load alters as a function of time. When combined with an underlying theoretical framework, for example ecological dynamics [118], physical and skilled characteristics could together be analysed to potentially provide rich insights into Australian football training and matches.

### Basketball

While there are data describing physical characteristics from different basketball leagues [119], the description of external load at the highest professional level (NBA), is limited [62, 120]. Game positional data is only accessible through the NBA’s official optical tracking provider, Second Spectrum (Los Angeles, U.S), and there are strict rules for the use of data for publication [62]. Commonly, other tracking systems are used during practices (those pre-approved by the NBA and the National Basketball Players Association), which implies a lack of homogeneity and compromises the ability of practitioners to build complete external load profiles across practice and competition [121]. As such, basketball sport scientists, performance and medical staff face numerous challenges on a day-to-day basis when it comes to using tracking systems and how to best use the information.

Basketball is an intermittent sport that, due to the court dimensions, number of players, and the rules [e.g., ball possession time (24 s)], requires the player to perform repeated high-intensity actions, such as rapid changes of direction and cutting actions, changes of speed in short distances, contacts (e.g., post-ups, screens, box-out), or run-to-jump actions, occurring between different locomotor demands (e.g., standing, walking, running, sprinting). Likely heavily influenced by pre-existing research from other team sports, the most common tracking metrics studied in basketball have been total distance, relative distance (distance/duration), distance and/or time in speed zones (total, relative and percentages), high-intensity actions (usually referred as distance, time and/or counts of accelerations, decelerations, jumps) and peak velocity [119, 122]. Moreover, as in other team sports, the analysis of describing the most demanding scenarios, both through discreet or fixed-length time epochs and rolling average time epochs, is emerging [86]. However, the mentioned influence from other team sports reflects a certain lack of critical thinking in the analysis of basketball specifically.

High-speed, very high-speed running and sprinting distance are commonly reported at > 10 km^.^h^−1^, 18 km^.^h^−1^ [123], and > 24 km^.^h^−1^ [86, 122], respectively, in the literature; whereas, top speed reached by players reported in the literature is ~ 20 km^.^h^−1^ [119, 124]. However, different results in peak speeds have been shown at the elite level (e.g., NBA; unpublished data). Based on the limitation of court size and the subsequent shorter lengths of explosive efforts in basketball, practitioners should reconsider the selection of peak speed as a key metric for planning and/or monitoring in the decision-making processes. The lack of consensus, and the actual requirements of distances at different intensities, requires that the practitioners consider reviewing speed thresholds for sprinting and high- and very-high speed running in basketball, independently of references from other team sports. Data mining techniques have been used to determine sport-specific thresholds, including fitting Gaussian curves [125], *k*-means clustering [126], and spectral clustering [127]. Such methods warrant consideration in basketball. Given the difference in the size of the playing area, it is likely that speed thresholds lower than other sports may be more suitable for analysing tracking data in the context of basketball.

Measures of velocity change (i.e., accelerations and decelerations) are other commonly used metrics, however, there is a lack of clarification and consensus across different tracking systems and manufacturers on how signals are filtered, calculations performed, or which are the suitable thresholds for this sport. Regarding the latter, thresholds for LPS vary from <  > 2 m^.^s^−2^ [86, 123], while research utilising IMU has used <  > 3 and 3.5 m^.^s^−2^ for total and ‘at high-intensity’, respectively [119]. Similarly, there are differences across tracking systems as to whether acceleration and deceleration data are reported in counts, distances or time spent changing velocity. Alternatively, a simple method for averaging the acceleration and/or deceleration profile of a team sport has been proposed to overcome issues with using predefined thresholds with time-series data [128]. While this analysis was conducted on rugby league athletes, the authors discuss the importance of such movements to physical preparation and performance across a variety of team sports. While sport-specific research should be undertaken for basketball, given the court size, the nature of the rapid movements required and the importance of actions such as turnovers, cuts, close outs, or defensive shuffles, it appears these movements are vital for managing injury risk, planning and monitoring the training process, and quantifying competition characteristics. Which calculation to use will depend upon the use of the information by the practitioner; for example, the summated acceleration profile may be most relevant for description and monitoring; whereas, the count and distance covered accelerating may be useful in programming individual workouts in a rehabilitation process.

Quantifying overall external load using accelerometery technology has been become a key metric in basketball. Many manufacturers have their own version of accelerometer-derived load, although PlayerLoad™ may be the original and the most commonly used [7]. It is recommended that practitioners seek to understand how manufacturer-specific “load” metrics are calculated, not only in basketball but all sports using this metric, since the measurement, filtering processes, and threshold rules differ. For example, some manufacturers calculate “load” from three-dimensional accelerometery data, while others use two-dimensional LPS for the calculation. Additionally, Schelling and Torres (2016) showed that constraints such as number of players, opponents, and court dimensions (i.e., half-, full-court) influence the external load [124]. Such studies are relevant for the practitioners to understand how the manipulation of constraints affect external load. Another pertinent aspect in basketball is the impact of the vertical load (z-axis) in the total count of ‘load’. The nature of this sport implies vertical actions (e.g., shooting, blocks), an aspect that has not been commonly reported in the literature, probably due to the lack of studies validating the quantification of jumps (and landing impact) across different tracking systems.

The evolution of tracking systems and machine learning techniques is allowing greater precision in the detection of basketball-specific movements. At present, technical aspects such as types of shots (e.g., driving layup or floater, pull-up jumper, step back, catch and shoot), picks, posts, isolations, off ball screens, among others, are recorded during matches with optical tracking. This, in combination with the metrics quantifying physical characteristics, can become a powerful tool for the generation of new and powerful insights in the description, planning and monitoring of external load in basketball.

### Ice Hockey

Ice hockey is an intermittent, collision sport played on ice, characterised by high-intensity bouts of skating with rapid changes in speed and direction [129] and high technical demands, such as puck control, evading defenders, and body checking [130]. Players rotate on and off the rink in shifts, each lasting approximately 30 to 80 s, generally between 20 and 35 times across 60 min of game time [131].

At the highest professional level, the National Hockey League (NHL), the 82-game regular season is played with a game approximately every 2.25 days, prior to a post-season that can include an additional 28 games over 60 days [132]. Due to shift rotations, there is a wide range of individual game-time per player, with the total time on ice potentially varying from approximately 5 to 28 min for skaters (excludes Goaltenders). Given this variation in game participation, compounded by the rate of competition, monitoring individual external load with a team is a worthwhile application of athlete tracking.

In order to monitor external load, tracking technology should be validated for the distinctive requirements of this sport. Notably, describing the unique biomechanical challenges of ice-skating reveals different characteristics to running [132]. Recent research has deemed an accelerometer-derived measure a reliable quantification of on-ice external load in a closed-roof hockey arena [133]. This measure can also reliably distinguish between certain ice hockey-specific movements including: acceleration, top speed, shooting, and repeated shift timing [133]. Describing external load, stratified into sport-specific categories and/or metrics, can further the understanding of technical and physical characteristics of training and competition. However, such microsensor technology may not be permitted in official competition and therefore, practitioners may be required to integrate such systems from the training environment with the different solutions permitted in competition.

Describing the high-intensity characteristics of skating with validated tracking technology is useful for the physical preparation of such athletes. One study of 36 NHL players demonstrated an average of seven high-intensity bouts per minute required, with high-intensity (> 17 km^.^h^−1^) skating accounting for approximately 45% of total skating distance [131]. However, this distribution of skating intensity is different according to position. Defensemen and forwards accumulate a similar distance across a game but in a different manner; with defensemen skating significantly higher distances at lower velocity skating speeds and forwards covering more in higher velocity bands [131, 134, 135].

Given these positional differences, there is opportunity for tracking data to assist with planning appropriate training drills and sessions, both on a positional and individual level. The selection of suitable temporal durations for analysis and in turn, planning, should be considered by the practitioner based on training objectives. While game-time is structured by shifts with varying work-to-rest ratios, training drills may at times be more continuous in nature with all skaters participating on the ice. This warrants a critical appraisal to consider the most appropriate time-series analysis. For instance, given a forward could spend ~ 22.7 s of a shift in maximal or near-maximal skating [135], a similar time epoch (notably less than one minute) may be used to better understand skating intensity. In addition to understanding the intensity across positions, there is also a “special teams” component in ice hockey with “power play” and “penalty kill” periods. These passages may have implications for physical preparation, given the difference in the number of skaters permitted on the ice.

Using the playing environment to assess fitness parameters, rather than requiring additional time for isolated testing, is an appealing solution that tracking technology may assist with. Positioning systems may be capable of measuring on-ice sprint times in place of timing gates, although the context of the sprint should be considered, with the duration and movement complexity influencing the reliability of the measure [136]. The ability to repeatedly produce power is important to success and tracking systems may be able to objectively capture this ability [137]. This may be particularly valuable given that similar off-ice (i.e. land-based) measures do not necessarily relate to on-ice performance [137].

Tracking systems are still a relatively recent addition to ice hockey, with competition player and puck training introduced to the NHL in the 2019–20 regular season [138]. As such, there may currently be a paucity of tracking research within this sport, but numerous potential avenues to explore going forward. These include; the indication of fatigue based on drop-off in tracking outputs [136], assessing team pace of play using spatio-temporal possession data [32], quantifying the unique characteristics of the Goaltending position, and continuing to describe the characteristics of the game across different competitions, age groups, and genders.

### Netball

Netball is a dynamic, high-intensity intermittent court-based team sport [139, 140]. Netball has unique physical [141], technical [142] and tactical [143] characteristics due to rules restricting players to specific areas of the court based on seven distinct playing positions, moving only one step when in possession of the ball and releasing the ball within three-seconds of receiving it [144]. Unlike other team sports, netball is capped at 15 min quarters, unless an injury occurs and play is halted, whilst the clock is stopped. Profiling the physical characteristics of netball athletes has largely been confined to video analysis and wearable IMU, due to training and matches being held indoors at the elite level [139, 141]. Recent advancements in LPS have allowed the physical characteristics of elite netballers to be profiled.

A key consideration for practitioners working within netball is the positional differences. Playing position defines where the players can move on court; Goal Keepers (GK) and Goal Shooters (GS) are restricted to only one third of the court, compared to Centres, who can play in all thirds (except for the shooting circles). These large discrepancies in the space available for players to move within greatly impacts the physical characteristics of match-play. Centre court players (centres, wing attack [WA] and wing defence [WD]) consistently have greater external loads compared to GK and GS [139, 140, 145]. Positions also differ in the contribution of locomotor (e.g., jogging, walking, shuffling, running) and non-locomotor (i.e., catch, jump, rebound, guarding) activities to total match load [146]. Sweeting et al. (2017) found that the movement sequences of GD, GA and WA are the most closely related, with GS being highly dissimilar to all other positions [110]. Therefore, it is imperative that practitioners working with tracking systems in netball acknowledge the positional differences when considering metric selection and analysis.

The distinct movement patterns of netball is another consideration. The tracking system used will determine the metrics utilised by practitioners; ongoing developments and increasing availability of technologies, including LPS, allow for tracking locomotor characteristics indoors [126, 140]. Whilst total distance and average speed can be examined with these systems [140], practitioners should consider how this is accumulated and the non-locomotor movements that are unique to the sport. Walking with straight movement and neutral acceleration have been found to be the most prevalent movement features in international match-play [126], and change-of-direction has been identified as an important external load metric in professional netballers [140]. When considering accelerometer-derived ‘load’ metrics, off-ball guarding has the greatest amount of PlayerLoad™ per minute, compared to other non-locomotor movements [146]. Further, IMA-derived metrics can investigate the non-locomotor movements and are particularly important for specific positions. For example, GS covers the lowest total distance, but performs the greatest number of total jumps [140]. Despite their use in research, IMA-derived metrics are yet to be validated during netball match-play, which must be acknowledged and considered.

The interchangeability of different manufacturers and providers is an issue for practitioners working in netball. During competitive matches, LPS could be used but teams may not have access to the data or system during training and subsequently, rely on inertial sensors to capture external load. Whilst wearable technologies such as IMU may have suitability to detect the “off-ball” movements in netball, as described above, clarity is needed on how to utilise the aggregate outputs of these metrics, including PlayerLoad™ per minute, for the design of training [146]. For example, many actions, off and on ball, can comprise the same PlayerLoad™ per minute. Therefore, practitioners may look to a systems approach to examine the key performance characteristics (physical and skilled) that exist within netball [56].

Given the high frequency of skilled actions and scoring in the complex and dynamic sport of netball, opportunity exists for sport scientists to place physical characteristics into context by overlying rich technical and tactical data. For example, a work domain analysis method, as part of a systems approach, for netball was recently investigated [56]. Specifically, 19 values and priority measures of elite netball were identified, including; passing, scoring, cognitive measures of psychological flow, team structure and the use of tactical timeouts, alongside physical characteristics [56], highlighting the need to integrate data. For example, measuring the acceleration or angular velocity of a netballer, when coupled with applying defensive pressure on an opponent could be a rich source of information [126]. Similarly, netballers use a variety of coordination strategies to shape tactical and physical behaviours during turnovers [147]. Together, these studies present a complex systems approach to analysing netball athlete performance that could potentially give further context to existing metrics, on *how* and *why* activities take place. For example, rather than presenting data on total distance covered or the number of accelerations that take place in netball, practitioners could complement this physical data with tactical and technical data to give richer insights.

### Rugby Codes

All three rugby codes (league, union and sevens) are played on the same field dimensions and are characterised as high-intensity intermittent contact sports [148]. Yet despite these similarities, distinct differences exist between codes. Rugby union and sevens are played under similar laws, with different playing numbers (15 vs. 7), whereas rugby league is played with 13 players per team and extensively different laws [148]. The different laws and playing numbers of the rugby codes result in unique characteristics that should be considered by practitioners when determining the use of tracking systems data in each code. Here we will focus on some key considerations for rugby league.

#### Rugby League

Games of rugby league are played over two 30 to 40 min halves (depending on the level of competition), separated by a 10-min rest interval. At the professional level players cover between ~ 5367 to 7064 m, with ~ 335 to 563 m HSR distance, whilst also carrying out ~ 21 to 34 collisions, depending on playing position, within a match [149]. Given these physical characteristics, and specifically the associated physiological, biomechanical, and energetic cost of such contact elements on players [1], quantifying both locomotor and contact demands as part of the external load is vital.

Following the validation of a collision detection algorithm [150], the use of tracking systems data to quantify collisions has increased [151]. Hulin et al. (2017) found Catapult Optimeye S5 devices to be sensitive (97.6 ± 1.5%) to detecting collisions, and the overall accuracy to increase when low intensity (< 1 PlayerLoad™ AU) and short duration (< 1 s) collisions are removed [150]). The use of tracking systems to detect collisions in rugby league enhances the ability to consider the locomotor and collision characteristics concurrently as opposed to separate entities [152]. For example, when quantifying and monitoring the ‘peak demands’ of rugby league competition, practitioners should consider: (1) the concurrent collision count, during the duration specific peak locomotive periods, and (2) the concurrent average running speed of the duration of the specific peak collision periods, to appropriately prepare players for the periods of competition.

Due to the collision nature of the sport, and the spatial confinements, players regularly accelerate and decelerate at high intensities; given the metabolic cost of these movements [128], it is important that they are also quantified and monitored. A range of acceleration metrics are utilised in rugby league, with average absolute acceleration becoming increasingly popular, especially for the analysis of the peak characteristics [82, 128]. This is an important trend given that acceleration has been shown to occur separately to peak periods of speed and yet, are equally important to the match outcome [81]. The use of PlayerLoad™, and its variants, has also been proposed to capture the acceleration, deceleration and change-of-direction, as well as the contact load [150] and are widely used in practice [16, 153]. Interestingly, the variant capturing the slow component (< 2 m/s) of PlayerLoad™, known as PlayerLoad™ Slow, has been used in rugby codes as a measure of sport-specific low-speed activity (e.g., rucking) [154]. Such accelerometry-derived metrics are also useful to capture external load in indoor training environments, given it is common practice for rugby league teams to carry out contact training in specific ‘padded’ rooms, and GPS derived acceleration metrics cannot be used in such environments.

Additionally, the unique technical and tactical requirements of the different positions within a rugby league team [155] result in differences in the physical characteristics of match-play. A main difference between positions is in the playing time of the match; forwards have lower playing times than backs [149, 156], which consequently influences the physical characteristics and should be taken into consideration. Backs are reported to cover greater total distance than forwards [152], however studies report no differences in the average running speed of match-play, given the differences in playing time between positions [156, 157]. Therefore, practitioners are encouraged to not only utilise total distances, but also consider the intensity of the work given the differing playing time, via analysis of the average running speeds of match-play and the peak characteristics for position specific training practices. Importantly, differences in HSR, very HSR, and collisions are present between positions. Forwards cover less HSR distance compared to adjustable and backs, but carry out more collisions [149]. As such, tracking metrics may therefore be of differing value to practitioners when seeking to monitor the external load, and subsequent dose–response, across positions.

Finally, the tactical characteristics of rugby league should be considered alongside the physical, to enhance the application of tracking systems data and aid in training practices. Whilst competition is 80-min in duration, the match can be broken up into distinct phases of play given the rule of the set of six tackles: attack, defence and the attack-defence transition, as well as ball-in-play periods. By considering the physical characteristics within these periods of play, practitioners can work with coaches when planning and evaluating technical-tactical training. Distinct locomotor characteristics exist during attacking and defending phases, with greater average running speeds during defense, but greater HSR distance per minute during attack [74]. Further positional differences are likely due to the unique positional requirements such as ‘backs’ leading the kick chase or challenging for the ball during the attack-defence transition. Therefore, practitioners working with tracking systems data in rugby league should consider the nature of the sport (e.g., contact) and positional differences, alongside the tactical characteristics when collecting, analysing and interpreting data.

### Soccer (Association Football)

Soccer is an intermittent field sport played by two teams of 11 players (10 outfield plus a goalkeeper) over two continuous 45-min halves, separated by a 15-min half time period [158]. The sport may be viewed as an early adopter of tracking systems, with much of the early research conducted in the 1990s stemming from optical tracking (predominantly semi-automated camera systems) and GPS use, in competition and training environments respectively, in the men’s professional game [158]. Despite being permitted in professional competition from 2015, many teams prefer to restrict GPS use to training only, potentially due to the less invasive nature of optical tracking. Thus, practitioners in these environments often face the challenge of integrating tracking data in order to consistently describe, plan, and monitor across the season. While integrative equations have been proposed, these are tracking system-specific, as well as dependent on the pitch size of the data collection [64].

Physical competition characteristics vary by playing position, depending on a number of situational factors, including tactical decisions, team formation, opponents style of play and level of competition [159, 160]. Total distance during a professional men’s match ranges from 10 to 12 km, with central midfielders covering the highest distance (11,885 m) while central defenders and strikers cover the lowest (10,671 m and 10,790 m, respectively) [161]. High-speed running (> 5.5 m^.^s) constitutes on average 12% of the total distance, however wide players are seen to cover a greater contribution of their total distance at high speeds [162, 163]. Similarly, wide players compared to central also produce the highest acceleration efforts, which is important due to the greater energetic demand of these movements [164]. The unique demands of the goalkeeping position result in 50% less total distance than outfield athletes (4–6 km), with 98% of match time spent in low intensity movement [165]. However, tracking systems have recently aimed to quantify goalkeeper-specific movement demands that include the number of dives, jumps, and overall forward and lateral explosive movements.

While absolute totals are necessary to describe the sport’s characteristics and monitor individual external load, practitioners are encouraged to think beyond absolute values to help guide physical preparation. The most simplistic use of relative (per minute) metrics enables identification of athletes' ‘pacing strategies’ [70]. For example, elite outfield male soccer players will range between 102 and 118 m/min depending on playing position [166]. Similarly, Fereday and colleagues (2020) identified the relative total distance between 120 and 190 m/min across a range of rolling average durations in professional male soccer players [90]. Rolling averages have been suggested as a superior analysis method compared to discrete 5–15 min epochs, due to a 12–25% underestimation in peak running demands [87]. Utilising peak locomotor demands to design position-specific training drills is common in applied soccer practice in an attempt to simulate game intensity [18]. However, concern has been raised over the validity of this concept, given that peak demands do not occur concurrently across metrics and players [94]. Practitioners are therefore reminded that the training process is complex and no single metric or calculation can surmise the external load an athlete is subjected to.

Drills in soccer are often manipulated in their design via field size, the number of players, or work-to-rest ratios, in order to elicit particular intensities. Practitioners using tracking systems in soccer can analyse the data to quantify the effects of such manipulation. For instance, larger pitch size provide greater opportunity to sprint whereas, smaller pitch sizes may allow less exposure to high velocities but greater exposure to changes of direction, accelerations and decelerations [5]. In particular, small-sided games have garnered popularity as a training methodology in soccer, however they should be combined with other forms of drills due to limitations on reaching higher velocities. High-speed exposure has been particularly highlighted as important for performance and injury risk perspective in soccer and therefore, many practitioners use the objective characteristics of soccer drills in comparison to competition characteristics to monitor athlete speed exposure over time [167]. However, despite the widespread adoption of regular high-speed exposure in practice, along with experts' opinion behind the concept [168], further work is required to establish stronger evidence in support of injury protection properties against regular high-speed exposure.

While this focus on quantifying and planning training drill design from a physical perspective is important, the technical and tactical components of soccer are also key contributors to success. Therefore, coaches and practitioners strive to combine physical preparation with the tactical element. One method, tactical periodization, has become a popular training strategy [169]. This methodology stresses different physical and tactical elements in turn across the microcycle, whereby the main focus is soccer-specific training [170]. Furthermore, the coach’s style of play heavily influences physical characteristics in soccer, as in other team sports, with tactical periodization assisting in training exercise selection that represent the specific coach’s principles of play [170]. With many professional leagues competing multiple times a week, along with congested schedules at other levels of play, the taxing schedule adds an element of complexity regarding preparation and recovery. As such, combining physical and tactical goals into training drills and sessions provides a time-efficient approach that tracking systems can support.

While soccer research has historically focussed on male athletes, there has recently been an increase in the women’s game [171]. Whilst the volume and intensity of total distance in the women is similar to that of males (8–11 km total; 108–119 m/min) [171–173], male players perform on average 30% more high-intensity movements during matches [171]. Therefore, to ensure appropriate application of tracking data to inform training and match preparation, an understanding of the physical characteristics specific to the female athletes is required. Particularly important to practitioners working with tracking data in women’s soccer is the consideration of suitable speed thresholds, given that most research has been conducted on men. A Gaussian curve fitting approach was used with instantaneous velocity data from women’s soccer and other team sports, with the intersections between curves used to determine sport-specific speed thresholds [125]. However, concerns have been raised, including the appropriateness of the technique itself—as there is no evidence to suggest that the velocities within each zone follow a Gaussian distribution—as well as the dataset used, which was not from an elite female population [127]. Consequently, another group used the spectral clustering technique on a dataset from 27 female players across 52 international matches, which determined thresholds of 12.5, 19.0, and 22.5 km/h most suitable to denote high-speed, very-high-speed, and sprint categories, respectively, for elite women’s soccer [127].

## Conclusion

We have attempted to summarise and critically evaluate the different tracking systems used within team sports, along with the suitability of their derived metrics for specific team sports. In summary, tracking systems provide the collection of athlete external load data, whereby practitioners can use derived metrics to describe, plan, monitor and evaluate training and competition characteristics. The selection of these metrics, and the systems from which they are obtained, are dependent upon the context of the sport and will need careful consideration by practitioners. Similarly, given the increasing amount of data generated, the accessibility and affordability of technologies to capture athlete external load, practitioners need to be critical in considering the validity, accuracy and precision of each system, along with metrics that provide ecological validity. Given the speed at which new metrics are introduced and developed by manufacturers, practitioners are encouraged to critically evaluate the suitability of those, within their chosen sport and attempt to “peak under the hood” of what is happening within algorithms and how data are being processed.

With the rise in popularity of open-source programming languages, spatiotemporal data can now not only be aggregated into drills, rotation stints, quarters, halves or matches, but also into time sequences such as rolling averages, frequency domain analysis and time series approaches. The speed and access of these approaches can now allow practitioners to sync vision with external load data, examine tactical or collective behaviour, merge skilled actions into a time series and quantify the specific movements of acceleration and angular velocity. However, practitioners are encouraged to maintain critical thinking, with a healthy dose of scepticism and awareness of appropriate theoretical frameworks, where possible, when creating a new or selecting an existing metric to profile team sport athletes.

## Data Availability

Not applicable.

## Abbreviations

GPS: Global positioning systems
LPS: Local positioning systems
IMU: Inertial measurement units
NBA: National Basketball Association
NFL: National football league
HSR: High-speed running
IMA: Total inertial movement analysis
NHL: National hockey league
GK: Goal keepers
GS: Goal shooters
WA: Wing attack
WD: Wing defence
AU: Arbitrary units
